# First Insights into the Effect of Low-Dose X-Ray Irradiation in Adipose-Derived Stem Cells

**DOI:** 10.3390/ijms20236075

**Published:** 2019-12-02

**Authors:** Annemarie Schröder, Stephan Kriesen, Guido Hildebrandt, Katrin Manda

**Affiliations:** Department of Radiotherapy and Radiation Oncology, University Medical Centre Rostock, Suedring 75, 18059 Rostock, Germany; stephan.kriesen@uni-rostock.de (S.K.); guido.hildebrandt@uni-rostock.de (G.H.); katrin.manda@uni-rostock.de (K.M.)

**Keywords:** adipose-derived stem cells, low-dose radiation, mesenchymal stem cells, radiation therapy, regenerative medicine, X-ray

## Abstract

(1) Background: Emerging interest of physicians to use adipose-derived stem cells (ADSCs) for regenerative therapies and the fact that low-dose irradiation (LD-IR ≤ 0.1 Gy) has been reported to enhance the proliferation of several human normal and bone-marrow stem cells, but not that of tumor cells, lead to the idea of improving stem cell therapies via low-dose radiation. Therefore, the aim of this study was to investigate unwanted side effects, as well as proliferation-stimulating mechanisms of LD-IR on ADSCs. (2) Methods: To avoid donor specific effects, ADSCs isolated from mamma reductions of 10 donors were pooled and used for the radiobiological analysis. The clonogenic survival assay was used to classify the long-term effects of low-dose radiation in ADSCs. Afterwards, cytotoxicity and genotoxicity, as well as the effect of irradiation on proliferation of ADSCs were investigated. (3) Results: LD (≤ 0.1 Gy) of ionizing radiation promoted the proliferation and survival of ADSCs. Within this dose range neither geno- nor cytotoxic effects were detectable. In contrast, greater doses within the dose range of >0.1–2.0 Gy induced residual double-strand breaks and reduced the long-term survival, as well as the proliferation rate of ADSCs. (4) Conclusions: Our data suggest that ADSCs are resistant to LD-IR. Furthermore, LD-IR could be a possible mediator to improve approaches of stem cells in the field of regenerative medicine.

## 1. Introduction

In Germany, low-dose radiation therapy (LD-RT) is an approved and commonly used option in the treatment of degenerative bone and inflammatory diseases [1], such as heel spur and osteoarthritis [2,3,4]. The success of this therapy is due to the anti-inflammatory and immune modulatory response in humans to LD-RT [5,6,7] mediated by the inhibition of the inflammatory cascade. In detail, LD-RT lead to an increased apoptosis rate of monocytes and granulocytes [8,9], as well as the suppression of adhesion processes of those immune cells to the location of inflammation [10,11], whereupon inflammatory reactions are prevented. The newest epidemiological studies and in vitro analyses confirmed the most effective dose at 0.5 Gy [2,12,13]. Likewise, according to current mathematical models, the carcinogenic risk of radiation treatment in this dose range of 0.5 to 1.0 Gy can be disregarded, as it is far below that of spontaneously occurring carcinogenesis [14].

However, further analysis of Jiang et al. (2008) on much lower radiation doses revealed that radiation in the range below 0.1 Gy can act as growth-promoting stimuli in several normal human cell lines but not in leukemia and solid tumor cell lines in vitro and in vivo [15]. Liang et al. (2016) and Truong et al. (2018) confirmed those trends in fibroblasts and lung cancer cell lines later on [16,17]. Those findings lead to an important question for scientists in the field of regenerative medicine: Could we also induce the proliferation of mesenchymal stem cells by LD-RT below 0.1 Gy to enhance the success of several regenerative therapies? Liang et al. (2011) and Yang et al. (2017) were the first who detected proliferation stimulating effects in human and rat bone-marrow stem cells (BMSCs) after X-ray treatment with low doses only below 0.1 Gy [18,19]. Therefore, in the following this particular dose range below 0.1 Gy is referred to as low-dose (LD).

Within the last few decades, stem cell research has focused more and more on the use of multipotent stem cells isolated from fat tissue, called adipose-derived stem cells (ADSCs) [20,21]. The reasons are obvious. ADSCs and BMSCs bear similar phenotypes, but ADSCs are more easy accessible from fat tissue compared to the more invasive and severe pain-associated bone marrow harvesting [22,23,24]. Today, fat grafts are commonly used in a broad variety of applications in the field of regenerative medicine [20,25]. However, the low survival rate of 10% to 30% of transplanted cells is one of the most challenging problems and the main reason why fat grafting procedures need to be repeated several times, until the result is satisfactory [26,27,28]. An improvement of this technique has already been achieved by enriching the stem cell fraction in the lipograft, called cell-assisted lipotransfer (CAL), but a sufficient result has not yet been achieved [29,30,31]. Promoting effects on the proliferation of tissue-specific stem cells by LD radiation could counteract this debacle. Therefore, the aim of this study was to analyse the proliferation-enhancing effect of low-dose irradiation (LD-IR) in adipose-derived stem cells. Likewise, various unwanted cytotoxic and genotoxic side effects are to be investigated. Since there is no consistent definition of low doses in the literature, for this study using exposure with X-rays LD-IR was defined using doses of ≤ 0.1 Gy, according to the Strategic Research Agenda of the Multidisciplinary European Low Dose Initiative (MELODI) when considering cancer risks [32].

## 2. Results

For this study, ADSCs of 10 different healthy female donors were obtained from human reduction mammoplasties and pooled to avoid donor-specific effects, as well as to enhance the experimental reproducibility. In our previous work we have already demonstrated that the endpoints of radiobiological investigations in pooled ADSCs (pADSCs; *N* = 10) are comparable to the average of the single analysis of each donor [33]. Due to slight differences in the growth rate of ADSCs of different donors, a relatively high number of 10 donors were chosen for pooling and experiments were performed only a few doubling times after cell pooling as described before [33]. Moreover, the isolated cells were characterized by the minimal criteria for the declaration of multipotent mesenchymal stromal cells [33,34], defined by the position statement of The International Society for Cellular Therapy in 2006 [33,34,35].

### 2.1. Low-Dose Radiation Does Not Induce Cell Damage

Several research groups have already demonstrated that medium (>2 Gy) and moderate radiation doses (0.5 Gy) induce negative side-effects in ADSCs regarding the cell proliferation and survival fraction [33,36,37], but to date the impact of low radiation doses (≤0.1 Gy) remain unclear. For this reason, we first examined the side-effects in ADSCs after LD-IR.

#### 2.1.1. X-Ray Irradiation Effects the Long-Term Survival of pADSCs Discontinuosly

**Figure 1:**
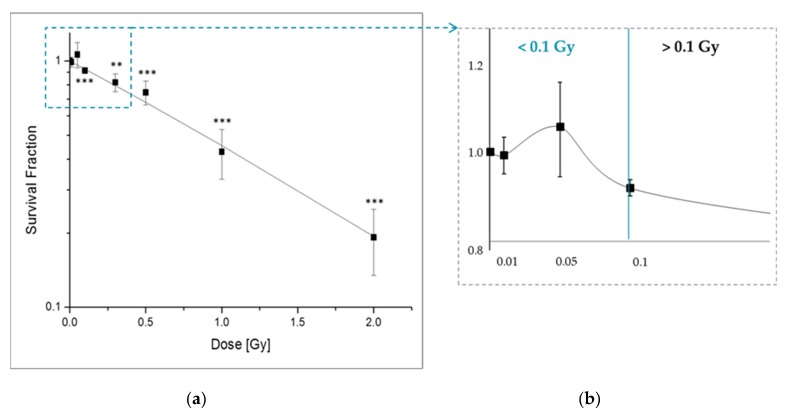
Clonogenic survival of pooled adipose-derived stem cells (pADSCs) after X-ray irradiation. pADSCs of 10 different donors were exposed to low (0.01, 0.05, and 0.1 Gy) and moderate (0.5, 1.0, and 2.0 Gy) doses of X-rays. Twenty days later, the cells were stained by crystal violet to visualize formed colonies. The cell survival fractions were normalized to those of sham irradiated cells. (**a**) Data from five independent experiments are presented as mean values ± SEM of the survival fraction. The significances refer to the 0 Gy control. Asterisks illustrate significances: ** *p* < 0.01, *** *p* < 0.002 (one sample *t*-test). (**b**) enlarged view of the low-dose area.

The effect of LD-IR on the long-term survival of pADSCs was determined by the colony forming assay and compared to the values due to low and moderate irradiation (IR) (Figure 1). In general, we could detect 87 ± 7 colonies formed from 1000 originally seed cells. This results in a plating efficiency of 7.83% ± 0.51%. While higher IR doses (>0.1 Gy) lead to a dose-dependent decrease in the survival fraction of the pADSCs (Figure 1a), discontinuous effects occur after LD-IR values (Figure 1b). Moreover, the exposure to low IR doses of 0.05 Gy resulted in an increasing trend of pADSCs survival by about 6%, whereas doses at 0.1 and 0.5 Gy already reduced this value by 9% and 15%. Furthermore, exposure to medium IR resulted in a strong reduction of the surviving fraction of 60% (1.0 Gy) and 80% (2.0 Gy). These results are comparable to further studies analysing the effect of IR on ADSCs, which however, used only 0.5 Gy as the smallest dose [33,37]. In contrast to IR in the high dose range with a clear dose-response relationship, after irradiation in the low and medium dose range non-linear dose-response relationships are already known from a lot of studies [38].

In the range of LD, we detected slight hyposensitivating trends—Also called increased radioresistance (IRR). Typically, IRR is observed in mammalian cells at a dose range between 0.3 and 0.6 Gy [39]. Here, we observed this in cells exposed to 0.05 Gy (Figure 1b). Reflecting the current state of science, IRR is associated with the transition from increased radiosensitivity (IRS) to IRR when a certain cell damage threshold is reached to activate DNA repair mechanisms [39]. However, this explanation, referred to as the Joiners-induced repair model [40], cannot be used in this case, because no previous dose range with hypersensitivity occurs. In addition, we detected increases in the survival fraction in ADSCs after LD irradiation, whereas previous descriptions of IRR within the literature took place in the low-dose range starting at 0.3 Gy. Rather, the data support the theory of low-dose induced hormetic responses in human cells describing beneficial low-dose effects of an agent that is harmful in high doses. Examples for such hormetic responses are the observed proliferation increasing effects of IR doses below 0.1 Gy in different non-carcinogen cell types [15,17,18,41].

Overall, however, in the low-dose range we did not detect any unwanted long-term effect on the survival rate of pADSCs after 0.05 Gy and only a small (9%) reduction after 0.1 Gy.

#### 2.1.2. No Cytotoxic Effects after IR with a Maximal Dose of 2 Gy

**Figure 2:**
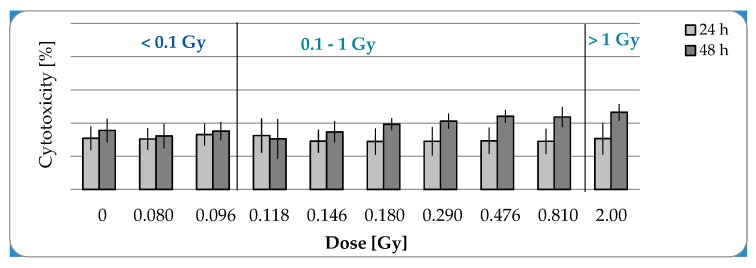
Lactate dehydrogenase (LDH) release as marker for cytotoxicity in pADSCs 24 and 48 h after X-ray irradiation. LDH activity was measured using the colorimetric LDH assay. The LDH release was normalized to this of the positive control. Data from three independent experiments are presented as mean values ± SEM (Mann–Whitney U Test).

In the further study, short-term effects of low and moderate irradiation doses were investigated. For this purpose the acticvity of Lactate dehydrogenase as a marker for cytotoxicity was measured in the culture supernatant of pADSCs, 24 and 48 h after irradiation procedure. No cytotoxic effects were detectable in pADSCs after exposure to LD-IR, whereas slight not statistically significant increase of approximately 5% was detected 48 h after exposure to moderate IR in comparison to the value 24 h after irradiation (Figure 2).

#### 2.1.3. LD-IR Neither Induces Residual nor Persistent DNA Double Strand Breaks in pADSCs

To assess IR-induced DNA damage within low and moderate doses, the frequency of DNA double strand breaks (DSBs) was verified, on the one hand, shortly after irradiation (0.5 h), to detect DNA damage, and on the other hand after an incubation time of 24 h after irradiation to analyze their repair.

**Figure 3:**
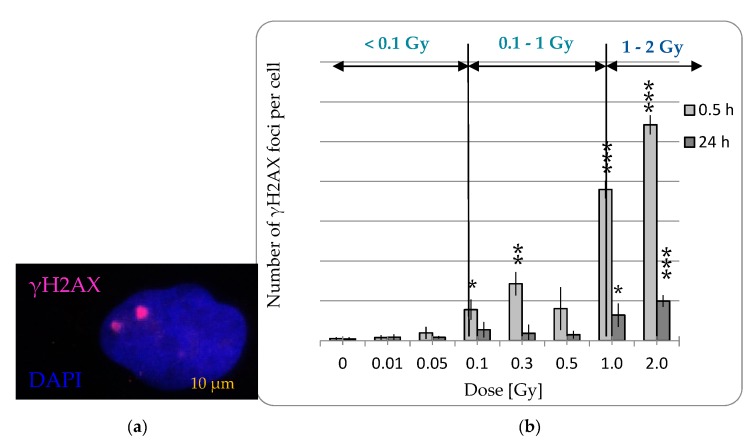
Capacity of pADSCs to repair DNA double strand breaks induced by irradiation (IR). The number of phosphorylated H2AX per nucleus was used to determine the corresponding number of DNA double strand breaks. To identify the DNA damaging effect of IR and the capacity of pADSCs to repair them, the cells were fixed 0.5 and 24 h after IR and incubated thereafter with anti-γH2AX antibody and IgG1 (red), as well as the DNA counterstaining with 4,6-diamidino-2-phenylindole (DAPI, blue). (**a**) Exemplary images of immune-cytochemistry staining after IR. (**b**) Results are illustrated as the mean number of γH2AX foci per cell ± standard deviation (SD) of three independent experiments; asterisks illustrate significant differences to * shame irradiated cells (control): * *p* < 0.05; ** *p* < 0.01; *** *p* < 0.001 (two-tailed *t*-test and two-way ANOVA with Bonferroni post-test).

In the range of doses <0.1 Gy neither residual nor persistent DNA DSBs were detected (Figure 3). Within this dose range the incubation time did not have an impact on the effect of radiation dose on the number of DNA DSBs (ANOVA: *p* > 0.05). Whereas low and moderate radiation doses led to a short-term accumulation of DNA DSBs (0.1 Gy: 7.76 ± 2.53, 0.3 Gy: 14.25 ± 2.92, 0.5 Gy: 8.02 ± 5.38), which in turn were repaired after a 24 h incubation period. From a radiation dose of 1 Gy, not only the induction of 37.96 ± 2.23 DNA DSBs was recorded, but also a rate of 6.39 ± 2.92 persistent DSBs. Therefore, from an IR dose of 0.1 Gy the number of DSBs increases significantly after a repair time of 24 h after irradiation (ANOVA: *p* < 0.01).

All in all, we could neither detect cytotoxic nor genotoxic effects in pADSCs after low doses <0.1 Gy.

### 2.2. Functional Changes

#### 2.2.1. Low Doses of X-Ray Enhances the Proliferation of pADSCs

As mentioned before, several studies have shown proliferation stimulating effects in different non-carcinogen cell types [15,17,18,41,42], but no data exist on those trends in ADSCs. Therefore, we also investigated the short-term effect of different radiation ranges in ADSCs.

**Figure 4:**
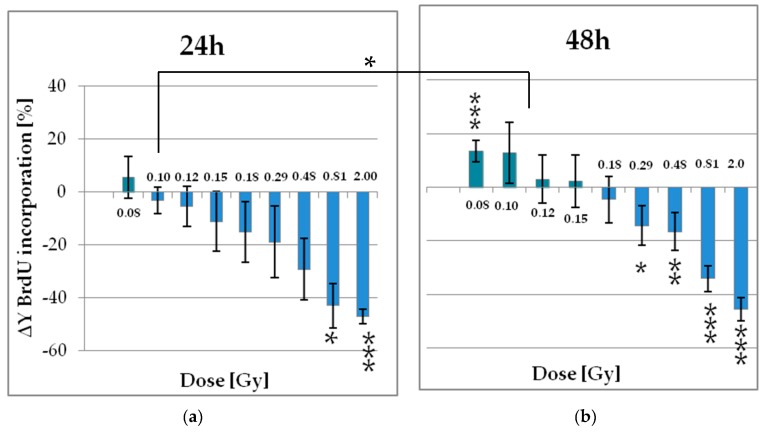
Alteration of the proliferation rate in pADSCs within 24 (**a**) and 48 h (**b**) following the irradiation procedure. Proliferation was determined by a colorimetric BrdU ELISA assay. Results are illustrated as mean ± standard deviation (SD) of five independent experiments. Asterisks illustrate significant differences referring to sham irradiated cells: * *p* < 0.02; ** *p* < 0.01; *** *p* < 0.002 (one sample *t*-test and two-way ANOVA with Bonferroni post-test).

As early as an irradiation dose of 0.29 Gy the proliferation rate was significantly reduced of about 14%. In contrast, LD irradiation 0.096 Gy and below increased this rate up to 15% (Figure 4). Medium doses even lead to a halving of proliferating cells. Those effects were not detected 24 h, but 48 h after irradiation. This time-dependency was only significant for the radiation dose of 0.096 Gy (ANOVA: *p* < 0.05). The growth kinetic of ADSCs is characterized by a relatively high population doubling time of about 60 to 90 h [33,43], depending on various donor factors such as age, body mass index (BMI), and others reviewed in [44]. Accordingly, the cell cycle was not completely traversed within these time periods of 24 and 48 h. Likewise, it can be assumed that the cell seeding procedure 24 h before IR has synchronized the pADSCs in the cell cycle to a certain extent. Therefore, one possible explanation could be that cells are affected by LD RT only in early cell cycle phases, whereas pADSCs of later phases that have already divided within the first incubation time of 24 h after IR are not affected. On the other hand, the reason for this time-depending variation could also be a time-depending amplification of the effect, so that during the first measurement period the experimental standard deviations are larger than the effects.

In general, however, it can be concluded that proliferation supporting effects are visible 48 h after IR, which relate to the population of irradiated cells, since the population doubling time has not yet been exceeded. As recognized before for the surviving fraction of ADSCs, ionizing radiation also induced hormetic effects within the LD range.

#### 2.2.2. MMP-2 Secretion of pADSCs is Enhanced after Low Dose X-Ray Therapy with 0.5 Gy

**Figure 5:**
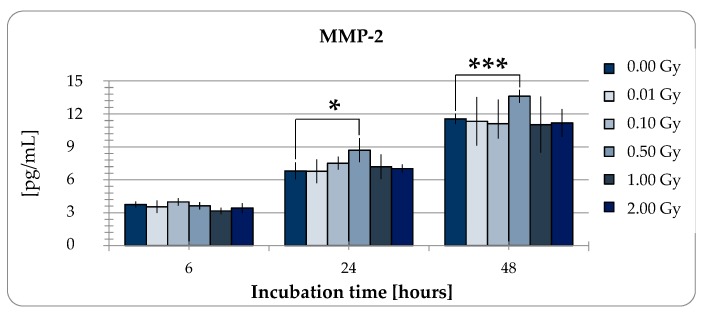
Content of matrix metalloproteinase-2 (MMP-2) in the supernatant of adipose-derived stem cells (ADSCs) 6, 24, and 48 h after irradiation. Results are illustrated as mean ± standard deviation (SD) of three independent experiments. Asterisks illustrate significant differences referring to sham irradiated cells: * *p* < 0.05; *** *p* < 0.005 (two-tailed *t*-test and two-way ANOVA with Bonferroni post-test).

ADSCs secretom plays a major role in angiogenesis, wound healing, tissue regeneration, and immunomodulation. Therefore, next to the proliferation capacity, we investigated the impact of LD-IR on the release of matrix metalloproteinases-2 (MMP-2) by ADSCs as it is an important mediator of the wound healing process in tissue repair. For this purpose, Enzyme-linked Immunosorbent Assays (ELISA) were used. Overall, pADSCs tend to a linear release of MMP-2 of approximately 6 pg/mL per day (Figure 5, ANOVA *p* < 0001). This rate (6 h: 3.75 ng/mL, 24 h: 6,81 ng/mL, 48 h: 11.56 ng/mL) was influenced neither by LD (6 h: 3.98 ng/mL, 24 h: 6.78 ng/mL, 48 h: 11.33 ng/mL) nor medium doses of 1 or 2 Gy ionizing radiation (6 h: 3.16/3.41 ng/mL, 24 h: 7.18/7.03 ng/mL, 48 h: 11.03/11.18 ng/mL), but increased by about 20% 24 and 48 h after 0.5 Gy irradiation treatment (6 h: 3.63 ng/mL, 24 h: 8.7 ng/mL, 48 h: 13.61 ng/mL). These data suggest that RT with 0.5 Gy that is typically used in an anti-inflammatoric manner could also improve the tissue repair mechansims induced by ADSCs. However, LD-IR did not reduce the release of MMP2 as a mediator for wound healing as its linear release was not disturbed.

## 3. Discussion

In the study presented here, the impact of low-dose of ionizing radiation (LD-IR) in adipose-derived stem cells (ADSCs) with respect to cyto- and genotoxic effects, as well as induced functional changes was investigated. Since there is no general definition of LD-IR in the literature, for this study LD-IR was defined using doses of 0.1 Gy or less. Since ADSCs are an easily accessible source of stem cells, the research has increasingly focused on them to improve the approaches of stem cells in the field of regenerative medicine. One aspect is to find a possible mediator to enhance the success of stem cells therapy. Here, we showed for the first time, that LD-IR promotes the proliferation of ADSCs. At the same time, we could exclude both cytotoxic and genotoxic side effects in those cells. We, therefore, propose LD-IR as a possible mediator to improve approaches of stem cells in the field of regenerative medicine.

In Germany, low-dose radiation therapy (LD-RT) is a commonly used option in the treatment of degenerative bone and inflammatory diseases [1] with most an effective dose at 0.5 Gy [2,12,13]. Previous cohort studies confirmed the positive effect of low-dose radiation on the well-being of patients, while at the same time an increased radiation carcinogenesis risk can be excluded [4,45,46,47]. For example, Zwicker et al. (2019) observed no additional cancer risk of female patients after radiotherapy of non-malignant disorders of the shoulder in comparison with the estimated spontaneous incidence of mammary carcinoma for this cohort [47]. Within this study the weekly single dose was 1 Gy and the median maximum dose 6 Gy [47]. According to the linear no-threshold (LNT) model, it can be assumed that the carcinogenic potential of those single doses of 1 Gy compared to doses smaller than 0.1 Gy is many times lower and can be neglected. Nevertheless, each hit of an 100 kV X-ray (1 mGy) brings about 150 reactive oxygen species as well as two DNA alterations, of which are about 10^−2^ double-strand breaks, and of 10^−4^ chromosomal aberrations [48]. As fundamental for the LNT-model, those rates increase proportional with rising radiation doses, as well as the resulting carcinogenic potential. Nevertheless, the calculated DNA damage induced by VLD is much less than the damage caused by the oxidative processes of normal metabolism [49,50]. However, the present data presented here of cell response to ionizing doses beyond 0.1 Gy does not fit into this model as cell damage is not increased in with LD irradiated cells and at the same time the proliferation is increased compared to shame irradiated cells. Those trends have also been recognized before in different non-carcinogen cell types [15,17,18,41] supporting the model of low-dose induced hormetic response. For example, in lung derived fibroblasts the exposure to radiation of 0.05 Gy stimulates the cell proliferation through transient activation of Raf (rapidly accelerated fibrosarcoma) and Akt (Protein kinase B) [41]. We detected the same proliferation stimulating trends in pADSCs, but after exposure of very small doses with 0.08 Gy. Furthermore, in our study we were able to determine for the first time increasing trends of the clonogenic survival of the ADSCs after LD-IR using 0.05 Gy.

In contrast, secretion of matrix metalloproteinases-2 (MMP-2) in pADSCs was increased only by the low-dose radiation of 0.5 Gy, whereas LD radiation resulted in no change. MMP-2 is an important mediator for wound healing processes of tissue repair reviewed in [47] suggesting that wound healing processes are enhanced after LD-IR. However, the group around Guo et al. (2010) have also observed positive effects on the wound healing of repeated low-dose radiation exposure of 0.075 Gy in a diabetic rat model, whereby an associated increase in bone marrow and circulating stem cells, vessel regeneration and cell proliferation in the wound tissue, and matrix metalloproteinase -2 and -9 expression was reported [42]. Therefore, despite our data, it cannot be ruled out that repeated exposure with LD has a positive effect on wound healing. Rather, this discrepancy shows the enormous need for research in this area and the enormous potential of LD-IR in the improvement of current stem cell therapies. We hypothesize that the increased proliferation of pADSCs may improve the success of regenerative therapies. However, this should first be examined in more detail. There are no in vivo studies on the effect of irradiating ADSCs below 0.1 Gy on the success of regenerative therapies. Guo et al. have already yielded promising results [42]. The underlying pathways and regulatory factors, however, remain misunderstood and should be subject of future investigations.

Taking into account that to date no negative side-effects were detected in cells after the exposure to radiation doses smaller than 0.1 Gy, relatively low-cost and harmless therapies could be conducted. For example, the success rate of autologous fat transplantation could be improved by promoting the proliferation of tissue-specific stem cells by LD radiation.

## 4. Materials and Methods

### 4.1. Cell Culture

#### Isolation of ADSCs

Human reduction mammoplasties were used to isolate ADSCs. The donors were healthy and female. This isolation was approved by the ethics committee at the University of Rostock, Germany (registration-number: A201008). The protocol used here has already been published in our previous work [34]. The ADSCs were cultured in Dulbecco′s modified Eagle medium and Kaighn′s modification of Ham′s F12 (DMEM/F12) supplemented with 10% fetal bovine serum Superior (FBS, Biochrom AG, Berlin, Germany) and 1% penicillin/streptomycin (P/S, 1%; 100 × penicillin 10,000 U/mL, streptomycin 10,000 µg/mL, Sigma-Aldrich, Steinheim, Germany). Reaching 80% confluence, the cells were washed twice with PBS and detached from the plastic surface of the culture flask with Trypsin/Ethylenediaminetetraacetic acid (Trypsin/EDTA, PAA Laboratories, Cölbe, Germany). A defined number of 500,000 cells in passage 1 were cryopreserved in DMEM/F12 containing 10% dimethyl sulfoxide (DMSO; Merck, Darmstadt, Germany) and 20% FBS.

### 4.2. Irradiation Procedure

The cells were irradiated with X-rays 24 h after seeding using the Xstrahl 200 therapy system (Xstrahl Ltd., Surrey, United Kingdom) at a dose rate of 0.52 Gy/min. Used single doses were 0.01, 0.05, 0.1, 0.3, 0.5, 1.0 Gy, whereas 0 Gy was utilized as the control and 2 Gy as positive control. To analyze a broad range of low doses within a single irradiation treatment, a shielding system was established using a MCP96-block (consisting of about 40% lead, 40% bismuth, and 20% tin) and a brass wedge. While the MCP96-block shielded the incident radiation to 100%, the brass wedge caused a shielding gradient with decreasing thickness from 96 to 50.5%. This arrangement was placed between the irradiation device and the cells cultured in a 96-well plate. At the single dose of 2 Gy applied here, the plate rows were thus irradiated between 0.08 and 2 Gy (Table 1).

### 4.3. Effects at the Cellular Level

#### 4.3.1. Colony-Forming Units Assay

The colony-forming units assay was used to analyze the long-term effect of IR on pADSCs cell survival. A number of 1000 pADSCs were cultured in T_25_ flasks (triplicates) and irradiated with different radiation doses (0, 0.01, 0.05, 0.1, 0.3, 0.5, 1.0, 2.0 Gy). Every three days, the medium was discarded and replaced by a fresh medium. After 20 days the cells were stained with crystal violet (1%, Serva Electrophoresis GmbH, Heidelberg, Germany). Visible colonies that contain at least 50 cells were counted using microscopy. The clonogenic survival fraction was calculated as follows:

(1) $$\mathrm{Plating}\;\mathrm{Efficiency}\;\left(\mathrm{PE}\right)\;=\;\frac{\mathrm{number}\;\mathrm{of}\;\mathrm{counted}\;\mathrm{colonies}}{\mathrm{number}\;\mathrm{of}\;\mathrm{seeded}\;\mathrm{cells}}\;\times \;100$$

(2) $$\mathrm{Survival}\;\mathrm{Fraction}\;=\;\frac{\mathrm{number}\;\mathrm{of}\;\mathrm{counted}\;\mathrm{colonies}}{\mathrm{number}\;\mathrm{of}\;\mathrm{seeded}\;\mathrm{cells}\;\times \;\left(\frac{\mathrm{PE}\;\mathrm{of}\;\mathrm{sham}\;\mathrm{irradiated}\;\mathrm{cells}}{100}\right)}$$

The calculated plating efficiency (PE) and survival fractions (SF) were evaluated using the data analysis and graphics software Origin 8.6.

#### 4.3.2. LDH Cytotoxicity Assay

The Lactate dehydrogenase (LDH) is a cytosolic enzyme present in mammalian cells. During the early phase of cell death, damaged plasma membranes release LDH into the cell culture media. Therefore, the LDH level in the cell culture supernatant is useful as a marker for cellular toxicity. Here, the LDH release after low-dose IR was measured by the colorimetric assay kit Pierce™ LDH Cytotoxicity Assay Kit (Thermo Fisher Scientific, Waltham, MA, USA). In brief, 24 h before irradiation treatment, 4000 pADSCs were seeded in quintuplicate into 96-well plates (TPP Techno Plastic Products AG, Trasadingen, Switzerland). At the time points of 24 and 48 h after IR, fifty microlitres of each sample or standard was mixed with 50 microlitres of reaction mix. For evaluating the positive control of 100% dead cells a triplicate of cells were treated with 10× Lysis Buffer 45 min before measurement procedure. Within an incubation period of 30 min at room temperature, the LDH present in the samples catalyzed the conversion of lactate to pyruvate via Nicotinamide adenine dinucleotide (NAD+) reduction to NAD-Hydrogen (NADH). Subsequently, this NADH was then used by diaphorase to reduce tetrazolium salt to red formazan product, which in turn was measurable at 490 nm. Optical densities were read using Anthos Zenyth 340 Plate Reader (Biochrom Ltd., Cambridge, UK).

(3) $$\mathrm{Cytotoxicity}\;[\%]\;=\;\frac{\mathrm{IR}\;\mathrm{treated}\;\mathrm{LDH}\;\mathrm{activity}-\mathrm{spontaneous}\;\mathrm{LDH}\;\mathrm{activity}}{\mathrm{Maximum}\;\mathrm{LDH}\;\mathrm{activity}-\mathrm{Spontaneous}\;\mathrm{activity}}\;\times \;100$$

#### 4.3.3. Cell Proliferation Assay

To determine changes in the number of proliferating cells, pADSCs were incubated with bromodeoxyuridine (BrdU, Cell Proliferation ELISA, Roche Applied Science, Mannheim, Germany) at 30 min prior to the IR procedure. BrdU is a synthetic nucleoside that incorporates into newly synthesized DNA of replicating cells, because of its similarity to thymidine. Therefore, the rate of incorporated BrdU can be used to assess the proliferation rate of cells. To analyze one generation of cells, incubation times that are lower than one population doubling time were chosen (96.5 ± 8.0 h [33]) of pADSCs.

24 h before IR 2000 cells were seeded in quintuplicate into 96-well plates. 1 h before irradiation, BrdU was added to the cells. After incubation times of 24 and 48 h the assay was performed according to the manufacturer′s instructions. Optical densities were read using Anthos Zenyth 340 Plate Reader. In order to calculate the relative BrdU incorporation, the measurement of unirradiated cells were defined as 100% BrdU incorporation. The BrdU incorporation as a direct marker for proliferation was calculated as follows:

(4) $$\mathrm{BrdU}\;\mathrm{incorporation}\;[\%]\;=\;\frac{\mathrm{OD}\;\left(\mathrm{sample}\right)-\mathrm{OD}\;(\mathrm{culture}\;\mathrm{medium})\;}{\mathrm{OD}\;\left(\mathrm{control}\right)-\mathrm{OD}\;(\mathrm{culture}\;\mathrm{medium})}\;\times \;100$$

(5) ΔY BrdU incorporation [%] = BrdU incorporation (control)−BrdU incorporation (sample)

### 4.4. DNA Damaging Effects—Measurement of DNA Doublestrand Breaks (γH2AX Assay)

The DNA damaging effect of IR procedure on pADSCs was analyzed using the γH2AX assay that visualize DNA double strand breaks within the nucleus. 24 h before IR, 35,000 pADSCs were seeded as duplicate in chamber slides (LabTek^®^, Nunc, Roskilde, Denmark), and treated according to the protocol already described [33].

### 4.5. MMP-2 Enzyme-Linked Immunosorbent Assays (ELISA)

10,000 pADSCs were seeded into each well of a 24-well plate and irradiated 24 h later. At the end points of 6, 24, and 48 h after irradiation, culture medium samples were collected, centrifuged, shock-frozen by means of nitrogen and stored at −80 °C until assayed. Concentration of matrix metalloproteinase-2 (MMP-2) was assayed using Human Quantikine ELISA Kit (R & D Systems, USA). Optical densities were read using Anthos Zenyth 340 Plate Reader.

### 4.6. Statistical Analysis

All data are presented as mean ± standard deviation (SD) or standard error of mean (SEM). The normality of the distribution of each parameter was assessed using the Shapiro–Wilk test. Non-normally distributed records were statistically evaluated by the Mann–Whitney U Test (LDH cytotoxicity assay). To identify differences between data sets, the two tailed students t-test was performed. To compare a variable under different conditions, here incubation time and irradiation dose (Cell proliferation assay, γH2AX Assay, and MMP-2 Enzyme-linked Immunosorbent Assay) the two-way ANOVA followed by a Bonferroni post-test with SigmaPlot (Version 13.0 from Systat Software, Inc., San Jose, CA, USA) was utilized. Significance was assessed at *p* < 0.05 (* *p* < 0.05, ** *p* < 0.01; *** *p* < 0.001). For comparative analysis of one dataset to a fixed value, as the survival fraction, the proliferation rate and cytotoxicity rate, the one-sample t-test was used. Here, a *p* < 0.02 was considered a significant difference (*: *p* < 0.02, **: *p* < 0.01; ***: *p* < 0.002).

## 5. Conclusions

Within the literature, there is a discrepancy in the definition of low radiation doses. Here, it has been shown that radiation treatment of ADSCs below 0.1 Gy leads to neither cytotoxic nor genotoxic changes. Rather, this radiation dose leads to increased proliferation, as well as elevated survival in ADSCs. The same was observed in several normal human cell lines, fibroblasts, as well as human and rat bone-marrow stem cells supporting the low-dose induced response model [15,16,17,18,19]. Therefore LD-IR could be a possible mediator to improve approaches of stem cells in the field of regenerative medicine as it supports the proliferation and survival.

## Figures and Tables

**Table 1:** Shielding of the irradiation dose by the used plumb block and the brass wedge.

Shielding by	Block	Brass Wedge								
Shielding [%]	*100*	96.0	95.2	94.1	92.7	91.0	85.5	76.2	59.5	0.0
Incoming radiation [Gy]	*0*	0.080	0.096	0.118	0.146	0.180	0.290	0.476	0.810	2.000

## Abbreviations

ADSCs	adipose-derived stem cells
Akt	Protein kinase B
BMSCs	bone-marrow stem cells
BrdU	bromodeoxyuridine
CAL	cell-assisted lipotransfer
DMEM/F12	Dulbecco′s modified Eagle medium and Kaighn′s modification of Ham′s F12
DMSO	dimethyl sulfoxide
DSBs	doublestrand breaks
EDTA	Ethylenediaminetetraacetic
ELISA	Enzyme-linked Immunosorbent Assay
FBS	fetal bovine serum
IR	Irradiation
LDH	lactate dehydrogenase
LD	low dose
LD-IR	Low-dose irradiation
LD-RT	low-dose radiation therapy
MMP 2	metalloproteinase-2
NAD	Nicotinamide adenine dinucleotide
NADH	Nicotinamide adenine dinucleotide-Hydrogen
pADSCs	pooled adipose-derived stem cells
PBS	phosphate-buffered saline
P/S	penicillin/streptomycin
Raf	rapidly accelerated fibrosarcoma
SD	standard deviation
VLD	very low-dose
